# A key role of GARP in the immune suppressive tumor microenvironment

**DOI:** 10.18632/oncotarget.9598

**Published:** 2016-05-27

**Authors:** Susanne A. Hahn, Annemarie Neuhoff, Jenny Landsberg, Jonathan Schupp, Daniela Eberts, Petra Leukel, Matthias Bros, Martin Weilbaecher, Detlef Schuppan, Stephan Grabbe, Thomas Tueting, Volker Lennerz, Clemens Sommer, Helmut Jonuleit, Andrea Tuettenberg

**Affiliations:** ^1^ Department of Dermatology, University Medical Center, Johannes Gutenberg-University, Mainz, Germany; ^2^ Institute of Translational Immunology and Research Center for Immunotherapy (FZI), University Medical Center, Johannes Gutenberg-University, Mainz, Germany; ^3^ Institute of Neuropathology, University Medical Center, Johannes Gutenberg-University, Mainz, Germany; ^4^ Department of Medicine II, University Medical Center, Johannes Gutenberg-University, Mainz, Germany; ^5^ Division of Gastroenterology, Beth Israel Deaconess Medical Center, Harvard Medical School, Boston, MA, USA; ^6^ Department of Dermatology, University Medical Center, Bonn, Germany

**Keywords:** Treg, melanoma, GARP, tumor microenvironment, tolerance

## Abstract

In melanoma patients, one of the main reasons for tumor immune escape and therapy failure is the immunosuppressive tumor microenvironment. Herein, suppressive immune cells and inhibitory factors secreted by the tumor itself play a central role.

In the present study we show that the Treg activation marker GARP (glycoprotein A repetitions predominant), known to induce peripheral tolerance in a TGF-β dependent way, is also expressed on human primary melanoma. Interestingly, membrane bound GARP is shed from the surface of both, activated Treg and melanoma cells, and, in its soluble form (sGARP), not only induces peripheral Treg but also a tumor associated (M2) macrophage phenotype. Notably, proliferation of cytotoxic T cells and their effector function is inhibited in the presence of sGARP. GARP expression on Treg and melanoma cells is significantly decreased in the presence of agents such as IFN-α, thus explaining at least in part a novel mechanism of action of this adjuvant therapy.

In conclusion, GARP in its soluble and membrane bound form contributes to peripheral tolerance in a multipronged way, potentiates the immunosuppressive tumor microenvironment and thus acts as a negative regulator in melanoma patients. Therefore, it may qualify as a promising target and a new checkpoint for cancer immunotherapy.

## INTRODUCTION

Malignant melanoma is the most common cause of skin cancer-related deaths worldwide. Therapeutic approaches include surgery, radiation, immunotherapy, chemotherapy, or their combinations.[1] Nevertheless, patients in advanced stages of disease have only few treatment options. One of the main reasons for tumor immune escape and therapy failure is the immunosuppressive tumor microenvironment.[2] Herein, inhibitory cell populations such as regulatory T cells (Treg), myeloid-derived suppressor cells (MDSC) and tolerogenic dendritic cells (tolDC), as well as inhibitory factors of the tumor itself play a major role.[3–5] Better understanding of inhibitory molecules and pathways regulating melanoma initiation and progression that could be used as therapeutic targets or biomarkers is needed.

Several studies have reported expression of Treg specific markers such as Foxp3 (forkhead-box-protein p3), CD39, or other stromal markers like CD73 in different tumor entities (e.g. pancreatic, gastric or lung carcinoma) correlated with lymph node metastasis and poor prognosis.[6–8] In the past, our group defined an important role for the soluble form of the Treg activation marker GARP (glycoprotein A repetitions predominant, sGARP), contributing to the induction of peripheral tolerance. sGARP was able to induce peripheral Treg with strong suppressive capacity in a TGF-β dependent way and prevented chronic inflammatory disease in a humanized mouse model.[9] Here, we investigated the role of GARP in the microenvironment of human melanoma in more detail. Our results revealed that several molecules such as Foxp3 and CD39/CD73 are shared by both, Treg and melanoma cells and that those Treg specific marker molecules are highly expressed on different melanoma cell lines compared to normal melanocytes. Surprisingly, we also detected GARP on the melanoma cell surface. sGARP not only directly regulates the function of CD4^+^ T cells resulting in the differentiation into induced peripheral Treg but also suppresses the effector function of CD8^+^ cytotoxic T cells and skews macrophages to an alternatively activated phenotype known to favor tolerance induction. Thus, the presence of GARP on melanoma represents a functionally relevant immunosuppressive mechanism contributing to peripheral tolerance by significantly preventing effector cell responses in the tumor microenvironment. Interestingly, treatment with one of the standard immunotherapeutic agents, IFN-α (Interferon-alpha), resulted in a decrease of GARP expression on both, Treg and melanoma cells demonstrating the clinical relevance of our findings. In conclusion, we suggest that Treg associated molecules expressed by tumor cells might have important roles in immune escape mechanisms and the inhibitory tumor microenvironment of melanoma. Furthermore, molecules shared by both melanoma and Treg might be ideal checkpoints and promising targets for novel immunotherapeutic strategies in cancer patients.

## RESULTS

### Regulatory T cells and melanoma cells share comparable inhibitory features

**Figure 1:**
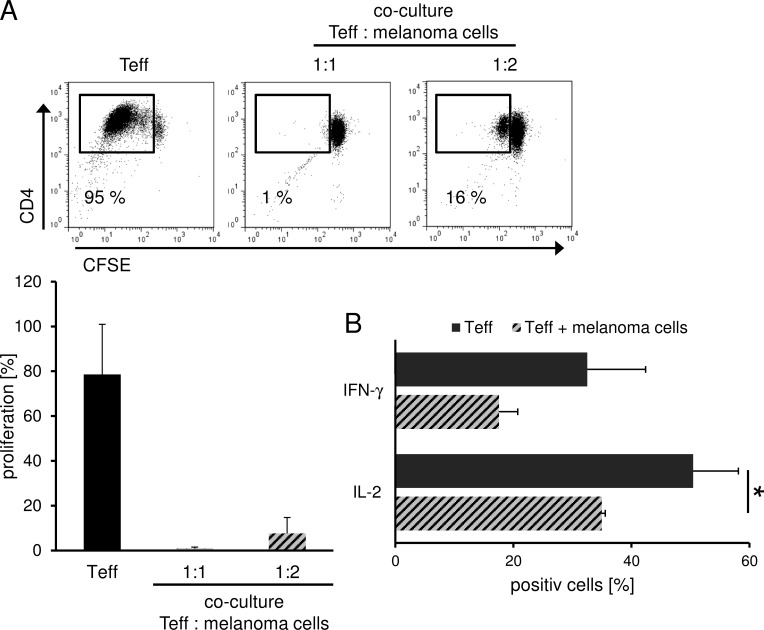
Treg and melanoma share similar inhibitory features Melanoma cells suppress T cell proliferation and cytokine production. **A.** CFSE-labeled CD4^+^ T cells were stimulated with anti-CD3 and anti-CD28 mAb (1 μg/ml each) alone or in co-culture with allogeneic SK29-MEL-1. Proliferation was analyzed on day 6 by flow cytometry using CFSE labeling. Numbers indicate percentage of proliferating cells. Dot plot show one representative result out of 5 independent experiments. Bar diagram shows pooled data (*n* = 5, means ± SD). **B.** Melanoma cell-mediated cytokine repression. CD4^+^ T cells were stimulated as indicated and intracellular cytokine staining of IL-2 and IFN-γ was performed 6 days after stimulation. Numbers indicate percentage of cytokine-producing cells. Bar diagram shows pooled data (*n* = 3, means ± SD, *P < .05).

The tumor microenvironment is known to promote immune escape mechanisms in melanoma. Herein, inhibitory cell populations such as Treg, myeloid derived suppressor cells (MDSC), tumor associated (M2) macrophages and tolerogenic DC (tolDC) play an important role.[4, 5, 10, 11] But also the tumor itself contributes to this phenomenon through the release of soluble factors such as TGF-β and IL-10 which then also induce immunoregulatory cells and inhibit T effector cell (Teff) function.[12, 13] In order to analyze the influence of melanoma cells on CD4+ Teff function, we performed allogeneic co-culture experiments and analyzed proliferation and cytokine production of Teff. Herein, we found a significant inhibition of T cell proliferation and effector cytokine production such as IFN-γ and IL-2 (Figure 1) resembling to the results obtained in co-culture experiments with human Treg as shown previously.[14, 15] Based on these data, we analyzed further potential phenotypical and functional similarities of melanoma cells and Treg.

### Treg markers are highly expressed on melanoma cell lines and primary melanoma in comparison to melanocytes

**Figure 2:**
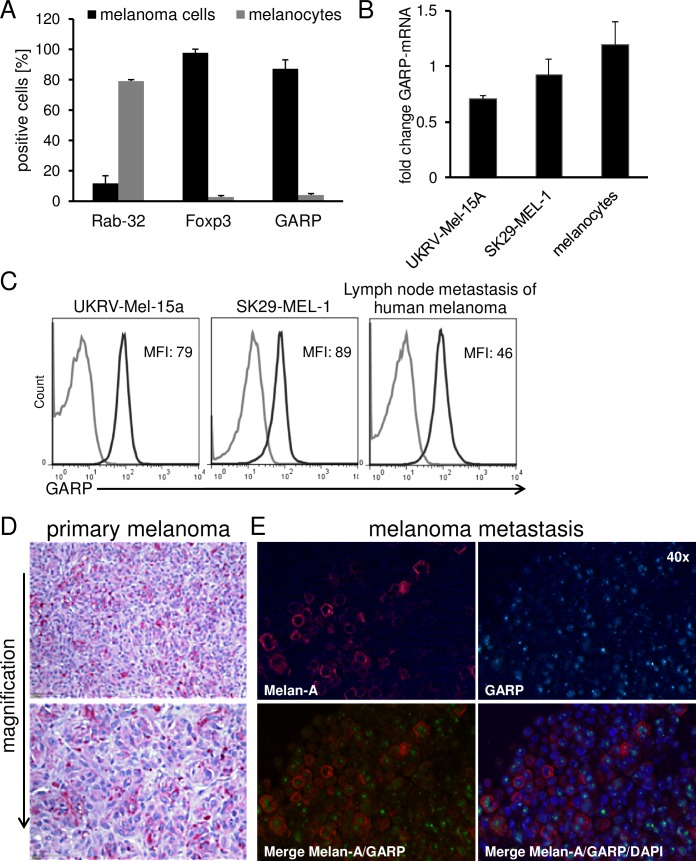
Treg marker expression on human melanoma cell lines and primary melanoma cells in comparison to melanocytes GARP is expressed on human melanoma. **A.** Flow cytometric analysis of Treg markers and Rab-32 on melanoma cells (SK29-MEL-1) and melanocytes. Cultured melanoma cells and melanocytes were stained and analyzed by flow cytometry. Bar diagram shows pooled data of 5 independent experiments (means ± SD). **B.** GARP-mRNA expression in UKRV-Mel-15A, SK29-MEL-1 and melanocytes was analyzed and normalized to the housekeeping gene ubiquitin C. Diagram shows fold change compared to the expression of activated Treg (*n* = 3, means ± SD). **C.** Flow cytometric analysis of GARP on different melanoma cell lines and cells derived from lymph node metastases. Single cell suspension from lymph node metastases were produced by gentle MACS dissociation, stained and analyzed by flow cytometry. Histograms show one representative result out of 5 independent experiments. Numbers indicate mean fluorescence intensities (MFI) of GARP minus isotype (black: GARP, grey: isotype). **D.** GARP expression was analyzed in paraffin embedded sections of primary melanoma. Shown is one representative result out of four independent experiments (four different patients). **E.** Immunofluorescence staining of Melan-A or GARP alone; corresponding merge of Melan-A, GARP and DAPI confirm co-localization of these proteins in melanoma cells of a melanoma brain metastases (using 40× objective).

As it has been described that several tumor entities are positive for Foxp3, CTLA-4 or CD39/CD73 [6, 16–18], all of them Treg associated markers, we reasoned whether also GARP, a recently described Treg activation marker involved in tolerance induction[9], is expressed on melanoma cells and would functionally contribute to the inhibitory features mentioned above. We found a significant expression of GARP and Foxp3 on melanoma cell lines SK29-MEL-1 (Figure 2A) and UKRV-Mel-15a, UKRV-Mel-21a, Ma-Mel-59a and Ma-Mel-91a (Suppl. Figure 1). Rab-32, a melanocyte marker associated to melanosomes and known to be lost during malignant transformation, served as control. Beside GARP expression on different melanoma cell lines demonstrating indeed a significant surface and as well as mRNA expression (Figure 2B), our results could be validated also in lymph node metastases of primary human melanomas as well as on primary melanoma sections by immunohistochemistry (Figure 2C-2E). Identified by size and morphology tumor cells were GARP positive, indicating that Treg marker molecules indeed may play a central role in human malignant melanoma. In order to detect co-staining of GARP and a melanoma specific marker we performed immunofluorescence staining on human melanoma brain metastases sections using primary antibodies against MelanA and GARP. Co-expression of GARP and MelanA could be shown in several melanoma cells (Figure 2E).

### GARP is shed from activated Treg and melanoma cells

**Figure 3:**
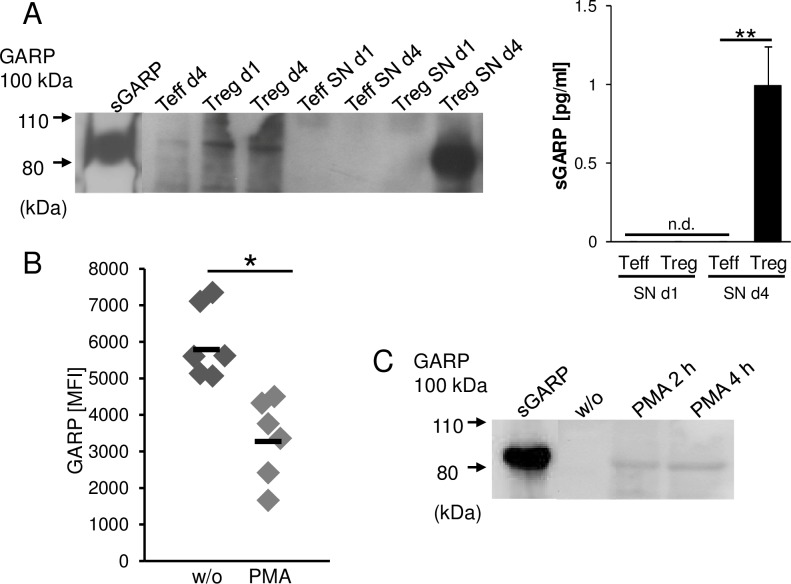
GARP is shed from activated Treg and melanoma cells GARP is found in Treg and melanoma supernatants. **A.** CD4^+^CD25^+^ Treg and CD4^+^CD25^−^ Teff cells were stimulated with anti-CD3 and anti-CD28 mAb (each 1 μg/ml). Supernatants (SN) were removed at different time points (day 1 and day 4) and cells were lysed and analyzed by western blot (left) or ELISA (right) using anti-GARP mAb. Recombinant sGARP was used as control. Figure shows one representative result out of four independent experiments (*n* = 4, means ± SD, **P < .01, n.d. = not detectable). **B.** Melanoma cells (UKRV-Mel-15A) were cultured with or without PMA (50 ng/ml) for four hours. GARP expression was analyzed by flow cytometry. Bar diagram shows one representative experiment out of 4 (*n* = 4, means ± SD, *P < .05). **C.** Supernatants were removed from melanoma cells cultured with PMA or untreated (w/o) at different time points and analyzed by western blot using anti-GARP mAb. Recombinant sGARP was used as control. Shown is one representative result out of three independent experiments.

Ectodomain shedding is a proteolytic mechanism by which transmembrane molecules are converted into a soluble form. This allows the cell to rapidly adapt a distinct surface phenotype and to generate soluble mediators that can act on other cells.[19, 20] To analyze whether GARP can be shed from Treg and melanoma cells and thus act as paracrine factor for the induction of peripheral tolerance, the supernatants of activated versus resting Treg were analyzed by western blot and ELISA (Figure 3A). sGARP was detected as early as four days after activation in supernatants of Treg, while the supernatants of Teff remained free of sGARP. A similar phenomenon was observed when GARP was studied in melanoma cells. GARP+ melanoma cell frequencies decreased after treatment of cells with PMA (Figure 3B) which is known to stimulate the release of transmembrane proteins [21] and in parallel, sGARP appeared in supernatants with a maximum at 4h as detected via western blot (Figure 3C).

### Soluble GARP influences phenotype and function of cells of the monocytic-myeloid lineage

**Figure 4:**
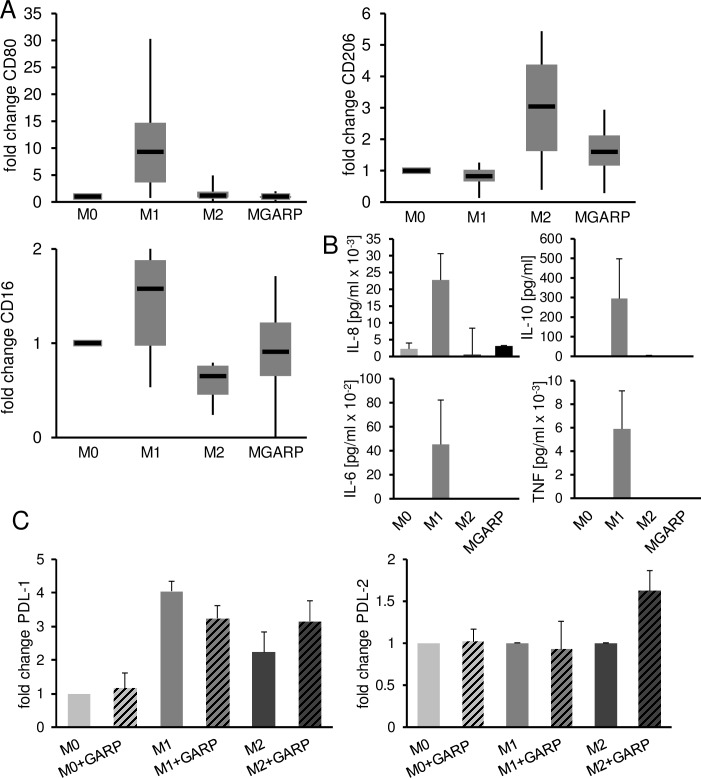
sGARP skews macrophages to an alternatively activated phenotype GARP affects the polarization of macrophages. **A.** CD80, CD206 and CD16 expression on M0, M1, M2 and MGARP macrophages. M0 macrophages were polarized into M1, M2 and MGARP (M0 stimulated with 10 μg/ml sGARP for 48 h) and analyzed by flow cytometry. Diagrams show fold change of MFI compared to the expression by undifferentiated (M0) macrophages (set to 1). Box blots show median with upper, lower quartile and minimum, maximum value (*n* = 6). **B.** Cytokine expression of M0, M1, M2 and MGARP macrophages analyzed *via* cytometric bead array. Bar diagrams represent pooled data of 4 independent experiments (mean ± SEM). **C.** PD-L1 and -L2 expression on polarized macrophages. M0 macrophages were polarized into M1, M2. After 24 hours of polarization cells were cultures with or without sGARP (10 μg/ml) for an additional day. Diagrams show fold change of MFI compared to the expression by undifferentiated (M0) macrophages (set to 1). Bar diagrams show pooled data of 4 independent experiments (mean ± SEM).

Inadequate immunity that occurs in tumor environment is in part due to the presence of tolDC or M2-type tumor-associated macrophages (TAM). Recent work by Peng et al. describes the influence of TGF-β on macrophages showing that TGF-β inhibition can reprogram TAM towards an M1-like phenotype.[22] As sGARP works in part through the TGF-βRII, we analyzed the influence of sGARP on the differentiation and polarization of macrophages. M0 macrophages were cultured in the presence or absence of sGARP and the resulting phenotype, cytokine production as well as T cell stimulatory capacity were investigated. As shown in Figure 4A, culture of macrophages in the presence of sGARP prevented expression of the M1 markers CD80 and CD16 but resulted in up regulation of the characteristic M2 marker CD206. In addition, the cytokine profile of sGARP treated macrophages showed similarities to the profile of M0/M2 macrophages, both known to be endowed with tumor promoting properties (Figure 4B). To analyze whether sGARP is able to influence polarized macrophages, we cultured M0, M1 and M2 after differentiation in presence of sGARP. We did not observe an alteration on common markers (data not shown). However, we detected an increase of PD-L1 and PD-L2 expression in presence of sGARP on M2 whereas M1 showed a reduction of PD-L expression (Figure 4C). These results are important with regard to the role of PD-L in tumor progression.[23–25]

In conclusion, these data demonstrate that sGARP skews macrophages to an alternatively activated phenotype and could be involved at least in part in polarization of tumor-promoting M2 macrophages.

Beside TAM tolDC belong to the inhibitory cell populations responsible for tumor immune escape. Terminally differentiated DC (mDC) play a central role in the induction of anti-tumor immune responses. In contrast, immature DC (iDC) are mainly involved in peripheral tolerance by inducing peripheral Treg.[26] Since sGARP is released into the supernatant of both, melanoma cells and Treg, we next assessed the effect of sGARP on phenotype and function of mDC and iDC. The presence or absence of sGARP did not result in significant alterations in phenotype of iDC or mDC (Suppl. Figure s2). Investigating the T cell stimulatory capacity of terminally differentiated mDC cultured in the presence of sGARP we found only a slight, not significant reduction of T cell proliferation. These results were observed for both CD4^+^ (Suppl. Figure 2) and CD8^+^ T cells (data not shown) demonstrating that sGARP only marginally influences DC phenotype and function.

### sGARP inhibits effector cell functions in CD4^+^ and CD8^+^ T cells and induces Treg

**Figure 5:**
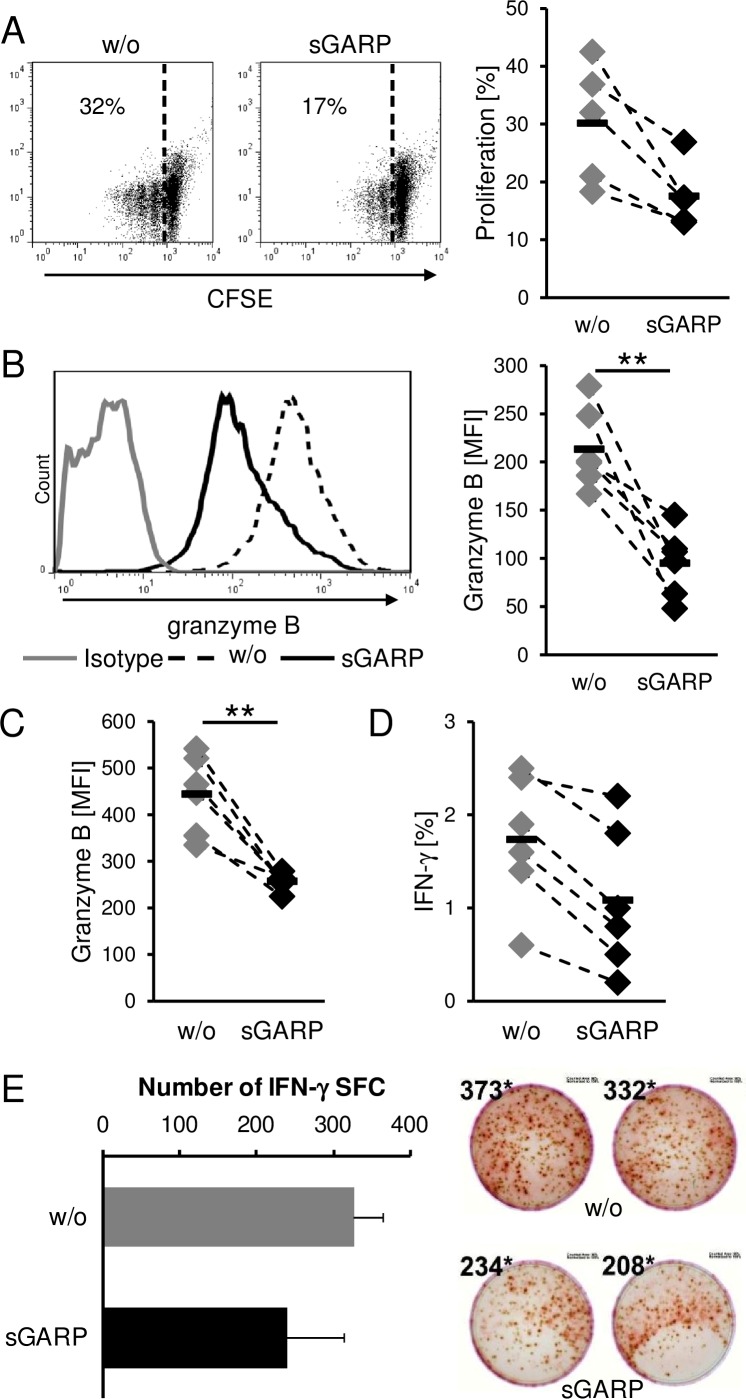
Suppression of cytotoxic T cell function sGARP diminishes proliferation and granzyme B expression in CD8^+^ T cells. **A.** CD8^+^ T cells were stimulated with anti-CD3 and anti CD-28 mAb (each 0.5 μg/ml) in the presence or absence of sGARP (10 μg/ml). To analyze the proliferation cells were labeled with CFSE. **B.** Granzyme B expression (% positive cells and mean fluorescence intensity) and (A) proliferation were analyzed on day 4 by flow cytometry. Dot plots show one representative result out of 5 experiments. Numbers indicate percentage of proliferating cells. Diagrams display summarized data of 5 independent experiments (*n* = 5, means ± SEM, *P < .05, **P < .01). **C.** Effect of sGARP on antigen specific CD8^+^ T cells. CD8^+^ T cells were cultured together with allogeneic melanoma cells for 7 days. 4 days after re-stimulation granzyme B expression and **D.** IFN-γ production were analyzed by flow cytometry. Diagrams display summarized data of 6 independent experiments (*n* = 6, means ± SEM, **P < .01). **E.** IFN-γ producing cells detected *via* ELISPOT. Diagrams display summarized data of 2 independent experiments (*n* = 2, means ± SEM, SFC, spot forming cells).

We detected high levels of GARP on melanoma cells, a marker thought to be strictly associated with activated Treg, as well as the presence of sGARP in the supernatant of activated Treg and melanoma cells. We already demonstrated that sGARP contributes to peripheral tolerance by inhibiting CD4^+^ T cell proliferation and effector cytokine production, by up regulation of Foxp3 and induction of peripheral Treg in a TGF-β-dependent way (Suppl. Figure 3). Therefore, in a next step we analyzed the functional relevance of this molecule addressing CD8^+^ T effector cells (CTL) that are key players in anti-tumor cytotoxicity. sGARP significantly reduced the proliferation of human CTL and significantly suppressed not only the frequency of granzyme B expressing cells but also the total amount (MFI) of granzyme B production (Figure 5A, 5B). This effect of granzyme B reduction in the presence of sGARP was also observed when CTL were stimulated with allogeneic melanoma cells. In agreement with these data, we detected a reduced antigen-specific IFN-γ production via flow-cytometry (Figure 5C, 5D) and ELISPOT (Figure 5E). In conclusion, sGARP modulates the antigen-specific CTL mediated immune responses through inhibition of important effector molecules such as granzyme B.

### Interferon-α significantly reduces GARP expression on melanoma cells

**Figure 6:**
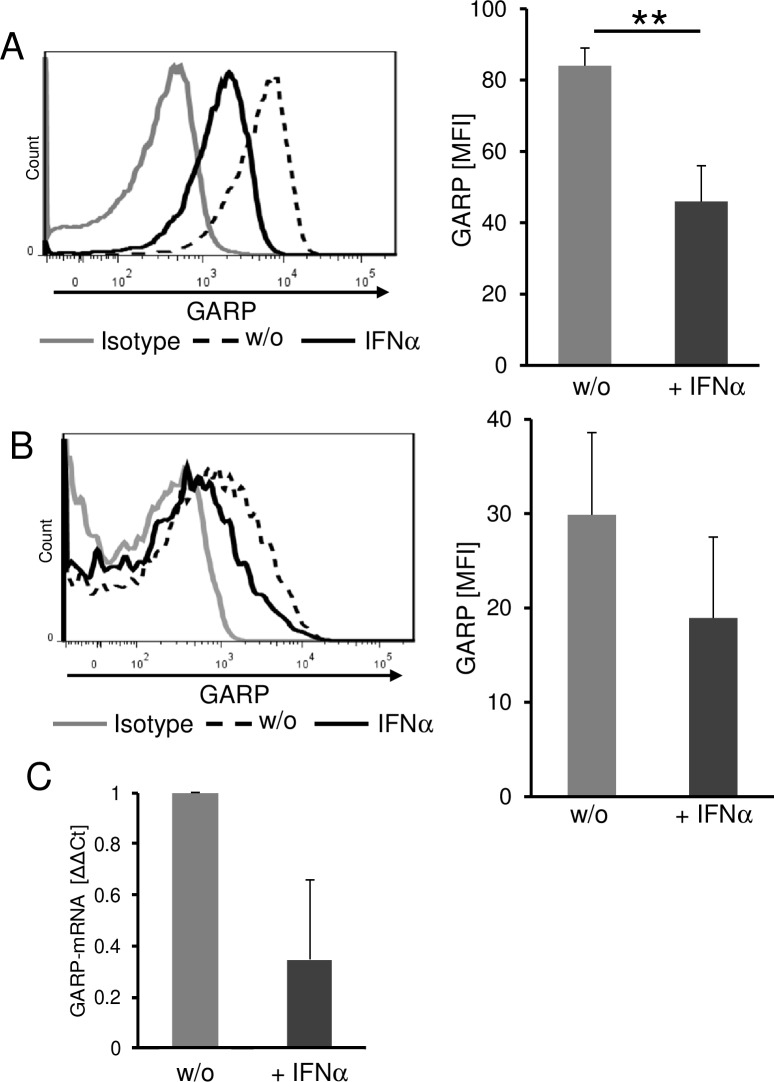
Interferon-α significantly reduces GARP expression on melanoma cells and on Treg Reduced GARP expression on melanoma cells and Treg in the presence of IFN-α. **A.** Melanoma cells were cultured for three days in the presence of 10^4^IU/ml IFNα and viable cells were analyzed by flow cytometry. The histogram shows one representative result out of 4 experiments. Bar diagram shows pooled data (mean ± SEM, **P < .01). **B.** CD4^+^CD25^+^ Treg were stimulated with anti-CD3 (0.5 μg/ml) and anti-CD28 (1 μg/ml) in presence of 10^4^IU/ml IFN-α. GARP expression was analyzed on day one by flow cytometry. The histogram shows one representative result out of 4 experiments. Bar diagram shows pooled data (mean ± SEM). **C.** GARP-mRNA expression in melanoma cells was analyzed after stimulation with IFN-α for 24 h and normalized to the housekeeping gene ubiquitin C. Diagram shows Δ-ΔCt of IFN-α treated cells compared to untreated (w/o = 1; *n* = 3, means ± SD).

In order to investigate the possible clinical relevance of our findings in more detail we analyzed the effect of immunomodulatory therapy on GARP expression using the example of IFN-α. IFN-α is one of the classical immunotherapeutic strategies used in the treatment of melanoma in an adjuvant setting.[27, 28] It has been described to exert beneficial immunomodulatory effects in Treg and melanoma cells.[29] Therefore, we analyzed the impact of IFN-α on GARP expression of melanoma and Treg. Melanoma cells cultured in the presence of IFN-α showed a significantly reduced GARP surface expression when compared to untreated control cells (Figure 6A). Furthermore, we could detect a reduced GARP expression in presence of IFN-α on activated Treg as well (Figure 6B). In order to exclude pro-apoptotic effects of IFN-α on tumor and T cells, we checked viability using 7-AAD (data not shown) and only viable cells were analyzed by flow cytometry. To foreclose the assumption, that there might be an increased shedding in presence of IFN-α we validated GARP expression on mRNA level. Results confirmed a remarkable decrease of GARP mRNA in melanoma after treatment with IFN-α (Figure 6C). These findings suggest that beside the various and well known effects on suppressing Treg, IFN-α also influences the immune inhibitory tumor microenvironment by decreasing GARP expression on melanoma cells and Treg.

## DISCUSSION

Malignant melanoma is one of the most aggressive malignancies in humans and is responsible for 60-80% of deaths from skin cancers. The 5-year survival of the majority of patients with metastatic malignant melanoma is poor despite success of new therapeutic strategies such as checkpoint inhibitory antibodies against CTLA-4 or PD-1 in a minority of patients.[30, 31] A major reason for tumor immune escape and therapy failure is the overwhelming immunosuppressive tumor microenvironment. Herein, inhibitory cell populations as well as inhibitory factors of the tumor itself play a central role. A better understanding of additional inhibitory molecules and pathways beyond PD-1/PD-L1 and CTLA-4 regulating melanoma initiation and progression that could be used as therapeutic targets or biomarkers is needed.

In the present study, we demonstrate that melanoma expresses the modulatory Treg marker GARP which contributes to an immunosuppressive tumor microenvironment. Additionally, we could detect sGARP in supernatants of activated Treg and melanoma cells. We show that sGARP promotes the polarization into M2 like tolerogenic macrophages and reduces the antigen-specific cytotoxic effect of CD8+ T cells by inhibiting their proliferation and granzyme B expression. These results demonstrate the contribution of GARP to the immunosuppressive tumor microenvironment. Interestingly, on mRNA level, GARP is also detectable in melanocytes, whereas surface expression is not increased. Whether GARP contributes to malignant transformation has to be investigated in future studies.

Structure-function analysis of GARP revealed that the extracellular but not the cytoplasmic domain is important for its function and binding capacity.[32] In the present study, we detected functional sGARP in the supernatant of activated Treg. Notably, we also showed that melanoma cells express and shed GARP from their cell membrane. Notably, human cancer cells frequently adopt a shedding strategy to generate soluble inhibitory molecules in order to circumvent tumor immunity. Different mechanisms can lead to shedding of extracellular domains of cell surface molecules, such as the enzymatic activity of ADAM (a disintegrin and metalloproteinase) proteins, and it remains to be shown what mechanisms are involved in the generation of sGARP.[20] So far the membrane protein GARP has not been detected on other tumor cells, whereas recent transcriptome analysis suggested expression in both, breast and prostate primary tumors and cell lines [33, 34].

It has been shown that GARP binds latent TGF-β and the function of sGARP is TGF-β receptor dependent and leads to Smad2/3-phosporylation.[9] TGF-β1 influences a variety of immune cells and consequently these cells express TGF-β receptors, with the TGF-βRII playing a central role in signaling.[35] Apart from having identified sGARP as important promoter of Treg function and regulator of peripheral T helper cell activity, [9] sGARP is a product of Treg and melanoma cells themselves (the present study). Here we show that sGARP influences several immune cells on different levels. CD8^+^ T cells are an important cell population for immune regulation and cytotoxic anti-tumor immunity. It has been described that TGF-β1 inhibits the expression of cytolytic gene products in CD8^+^ T cells.[36] Accordingly, in our study sGARP reduces the production of the cytolytic protein granzyme B and diminishes the proliferation of CD8^+^ T cells, suggesting that sGARP is an important antagonist to cytotoxic T cell function in the tumor microenvironment.

DC are an important tool for tumor-antigen-specific immunotherapy of cancer because of their central role in the initiation of immune responses. DC vaccination using tumor-antigen-loaded DC leads to induction of tumor-antigen specific T cells even in advanced-stage cancer patients.[37] Furthermore, DC vaccination reduces Treg frequencies and effects the rehabilitation of impaired T-cell activity.[38] Nevertheless, despite these findings, objective clinical responses in these patients are rare. In the present study, we observed that sGARP only slightly diminished the stimulatory capacity of DC. Nevertheless, sGARP induced a tolerogenic tumor micromilieu also affecting the function of antigen-specific T cells. Thus, T cells induced following DC vaccination are impaired in their activity in the presence of sGARP, explaining in part the failure of DC vaccination with regard to objective clinical responses in advanced-staged melanoma patients even though antigen-specific T cells can be found in peripheral blood of tumor patients.

Beside the influence of sGARP on DC and CD8^+^ T cells we analyzed the effect on macrophages. Notably, sGARP induced M2 polarization in M0 macrophages. While the function of macrophages does not always match phenotypic characteristics and their subdivision into M1 or M2 cells, tumor associated M2-phenotype macrophages were generally shown to inhibit function of immune cells and promote tumor cell survival.[39–41] This implicates sGARP as a novel soluble factor that favors (functional) M2 polarization which additionally supports an immune suppressive tumor microenvironment.

A further hint to the clinical relevance of GARP is its reduced expression on Treg as well as on melanoma cells after treatment with IFN-α. Thus GARP not merely suppresses different immune cells in the tumor microenvironment, but is also regulated by the clinically approved and in the adjuvant setting routinely used immunotherapeutic drug IFN-α. Further studies now have to show whether serum levels of sGARP are detectable in sera of melanoma patients and eventually correlate with advanced disease stages.

Taken together, our data indicate a key role of GARP in the tumor microenvironment, inducing and promoting tumor immune tolerance via multiple pathways. Moreover, since GARP is not only expressed by activated Treg but also by melanoma cells, it may be an ideal target for a combinatory tumor immune therapy and serve as a novel checkpoint in future therapeutic strategies.

## MATERIALS AND METHODS

### Melanoma cell lines and their stimulation

The human melanoma cell lines SK29-MEL-1 and UKRV-Mel-15A were described previously.[42, 43] Cells were obtained from Prof. Dr. T. Wölfel, Mainz, Germany (SK29-MEL-1) and Dr. A. Paschen, Essen, Germany (UKRV-Mel and Ma-Mel) in 2014. Cell lines have been tested and authenticated at Leibniz Institute (DSMZ) in Braunschweig, Germany, using DNA profiling lastly in September 2014. Generated STR profiles were matching the STR reference profile of respective parental cell lines from cell banks ATCC, HPACC, JCRB, RIKEN, KCLB, EMBL and DSMZ. Briefly, cells were cultured in RPMI-1640 supplemented with 10% fetal calf serum. For some experiments melanoma cells were cultured together with PMA (phorbol 12-myristate 13-acetate; 50 ng/ml) or IFN-α (10^4^ IU/ml).

### Isolation and stimulation of human T cell populations

Buffy coats were obtained from healthy volunteers, with approval by the local ethical committee (Landesaerztekammer Rheinland-Pfalz). CD8^+^ T cells, CD4^+^CD25^−^ and CD4^+^CD25^+^ T cells were isolated as previously described.[14, 44, 45] For some experiments, peripheral blood mononuclear cells (PBMC) were depleted of T cells using CD3-Dynabeads (0.5 beads/cell, Invitrogen).

For proliferation assays, cells were labeled with carboxyfluorescein succinimidyl ester (CFSE) and cultured in 48 well plates at 10^6^ cells/ml, stimulated with 0.5 μg/ml anti-CD3 mAb (clone OKT3) plus 1 μg/ml anti-CD28 mAb (clone 28.2, eBioscience) in presence or absence of different ratios of melanoma cells.

To analyze the effect of sGARP on proliferation and granzyme B expression of CD8^+^ T cells, T cells were stimulated with anti-CD3 mAb (0.5 μg/ml) and anti-CD28 mAb (1 μg/ml) or with melanoma cell line D05-MEL#6 (5 × 10^4^/ml) in presence or absence of sGARP (10 μg/ml) from R&D Systems (#6055-LR, <0.01 endotoxin units per 1 μg) as shown before.[9] At day 7, T cells were harvested and re-stimulated with melanoma cells or anti-CD3 mAb (0.5 μg/ml) plus irradiated (90 Gy) T cell-depleted PBMC. For some experiments, T cell proliferation was measured by additional 16h-pulse with ^3^H TdR (37 kBq/well) using a liquid beta-scintillation counter.

### Enzyme-linked immunosorbent assay

GARP in supernatants was analyzed by ELISA according to the manufacturer's protocol (BioLegend, #440107).

### IFN-γ ELISPOT assay

CD8^+^ T cells (5 × 10^5^/ml) were isolated and co-cultured together with allogeneic melanoma cell line D05-MEL#6 (5 × 10^4^/ml) in presence or absence of sGARP (10 μg/ml) in AIM-V/10% HS containing 25 IU/ml IL-2 for 7 days. Four days after re-stimulation under the same conditions (CD8^+^: 4 × 10^5^/ml, D05 Mel: 5 × 10^4^/ml) IFN-γ ELISPOT assays were performed and evaluated as described.[46]

### Western blot

Lysates and supernatants in NuPAGE LDS buffer were submitted to SDS-PAGE and western blot with anti-GARP (Enzo Life Sciences, #ALX-804-867). Primary antibody reactivity was detected using anti-mouse-HRP followed by chemiluminescence.

### Real-time PCR

Total RNA was extracted from 10^6^ cells using the RNeasy^®^ Plus Mini Kit (Qiagen) according to the manufacturer's instructions. cDNA was generated by reverse transcription using the iScript™ cDNA Synthesis Kit (Bio-Rad). Gene expression levels were determined by quantitative qRT-PCR on the ABI 7300 Real Time PCR System (Thermo Fisher Scientific) using the Fast Plus EvaGreen® qPCR Kit (Biotium). mRNA expression of *GARP* (forward primer: 5′-CAC TGA CTG AGC TGG ACC TT-3′; reverse primer: 5′-CAA GAT TGA GCC GCT TGA GG-3′) was normalized to the expression level of the housekeeping gene *UBC* (ubiquitin C; forward primer: 5′-CCC CAG TAT CAG CAG AAG GA-3′; reverse primer: 5′-ATC GCC GAG AAG GGA CTA CT-3′) in each sample. Relative mRNA expression was calculated in reference to untreated samples using the Δ-Δ Ct method.

### Culture of dendritic cells and macrophages

To analyze the effect of sGARP on DC, PBMC-derived monocytes were isolated by plastic adherence and kept in X-VIVO-15 (Lonza) plus 1% heat-inactivated autologous plasma including 800 U/ml GM-CSF and 1000 U/ml IL-4. At day 5 non-adherent cells were rinsed off. For terminal differentiation into mature DC (mDC) the cells were additionally stimulated on day 6 with 10 ng/ml IL-1β, 10 ng/ml TNFα, 1 000 U/ml IL-6, 1 mg/ml PGE_2_ (prostaglandin E2),[26, 47] with or without 10 μg/ml sGARP. iDC (immature DC) were cultured for two days with or without 10 μg/ml sGARP. iDC and mDC were harvested at day 7 and used for T cell stimulation.

To generate macrophages, isolated monocytes were cultured in RPMI-1640 plus 1% heat-inactivated autologous plasma including 50 ng/ml M-CSF (macrophage colony-stimulating factor, M0-Medium). At day 6 cells were harvested and either used as un-polarized macrophages (M0) or transferred to 24-well-plates for polarization. For polarization, cells were exposed to fresh M0-Medium containing either 20 ng/ml IFN-γ + 100 ng/ml LPS (M1 polarized macrophages), 20 ng/ml IL-4 (M2 polarized macrophages), or 10 μg/ml sGARP (GARP polarized macrophages; MGARP) for an additional 48 h.

### Flow cytometry

Flow cytometric analyses were performed using the following antibodies: anti-CD4, anti-CD11b, anti-CD33, anti-CD83, anti-CD86, anti-CD206 (BD Biosciences), anti-CD14, anti-CD58, anti-CD80, anti-HLA-DR (ImmunoTools), anti-CD16 (Thermo Scientific), anti-CD25, anti-GARP (eBioscience) and anti-Rab-32 (Abnova).

For intracellular staining of Foxp3, cells were fixed and permeabilized using a Fix/Permeabilization kit (eBioscience) and stained with anti-Foxp3 mAb (BD Biosciences). Cytokine expression was analyzed in T cells re-stimulated with 50 ng/ml PMA plus 1 μg/ml Ionomycin for 5 h in the presence of Monensin (1.3 μM) 7 days after *in vitro* primary stimulation (day 0). Cells were then permeabilized as above and stained with anti-IL-2, anti-IFN-γ or anti-granzyme B mAb (BD Biosciences). Flow cytometry was performed on an LSRII FACS and FACSCalibur (BD Biosciences), using FlowJo software (Tree star).

### Cytokine quantitation using cytometric bead array (CBA)

Cytokine levels were analyzed in tissue culture supernatants using a BD CBA human inflammatory cytokine kit (BD Biosciences). The CBA kit simultaneously measures IL-1β, IL-6, IL-8, IL-10, IL-12p70 and tumor necrosis factor alpha (TNF-α). The assay was performed according to the manufacturer's instructions.

### *In situ* detection of GARP in human primary melanoma and melanoma metastasis

Immunohistochemistry was performed on 4 μm-thick routinely processed formalin-fixed and paraffin embedded melanoma sections with a biotinylated anti-GARP (Enzo Life Sciences, #ALX-804-867) primary antibody, followed by enzyme-conjugated secondary antibody and the LSAB-2 color development system (DAKO). Stained sections were examined with a Leica DMLB microscope; images were acquired with a JVC digital camera KY-75FU. Immunofluorescence staining was also performed on 4 μm-thick routinely processed formalin-fixed and paraffin embedded melanoma brain metastases sections. After antigen retrieval using Dako target retrieval solution (Dako #S2368) sections were stained with anti-GARP (Acris antibodies GmbH, #AP17415PU-N) primary antibody combined with anti-MelanA (Dako #M7196) primary antibody followed by fluorescence-labeled secondary antibodies Alexa Fluor 488 goat anti rabbit and Alexa Fluor 546 goat anti mouse (Invitrogen #A11034 u A11030). Nuclei were counterstained with DAPI (Thermo Scientific, #62248). Stained sections were examined with a Leica DM600B microscope, images were acquired at 40x magnification with a digital camera (MBF CX9000).

### Statistics

Results represent means ± SEM. Statistical significance was determined using the Student *t* test with *p<.05, **p<.01, ***p<.001 and n.s. (not significant) as indicated.

## SUPPLEMENTARY TABLES


