# Supplementary material for: A key role of GARP in the immune suppressive tumor microenvironment
Source: Oncotarget. 2016 May 27;7(28):42996–3009. doi: 10.18632/oncotarget.9598 (PMC5190003; doi:10.18632/oncotarget.9598)
Supplement: Supplementary file 1 [file oncotarget-07-42996-s001.pdf]

A key role of GARP in the immune suppressive tumor microenvironment

Supplementary Material

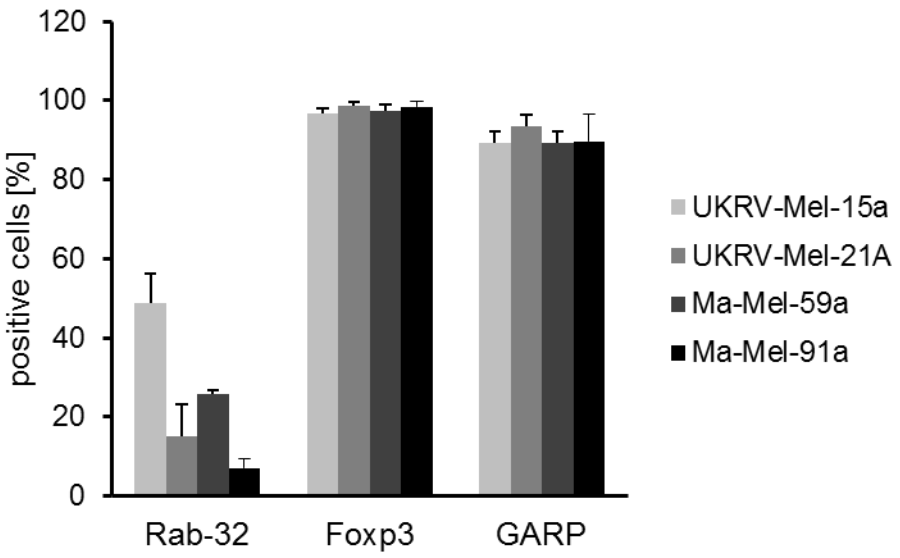

Supplemental Figure 1

GARP is expressed on different melanoma cell lines. Flow cytometric analysis of RAB-32, Foxp3 and GARP on melanoma cells: UKRV-Mel-15a, UKRV-Mel-21A, Ma-Mel-59a and Ma-Mel-91a. Cultured melanoma cells were stained and analyzed by flow cytometry. Bar diagram shows pooled data of 4 independent experiments (means ± SD).

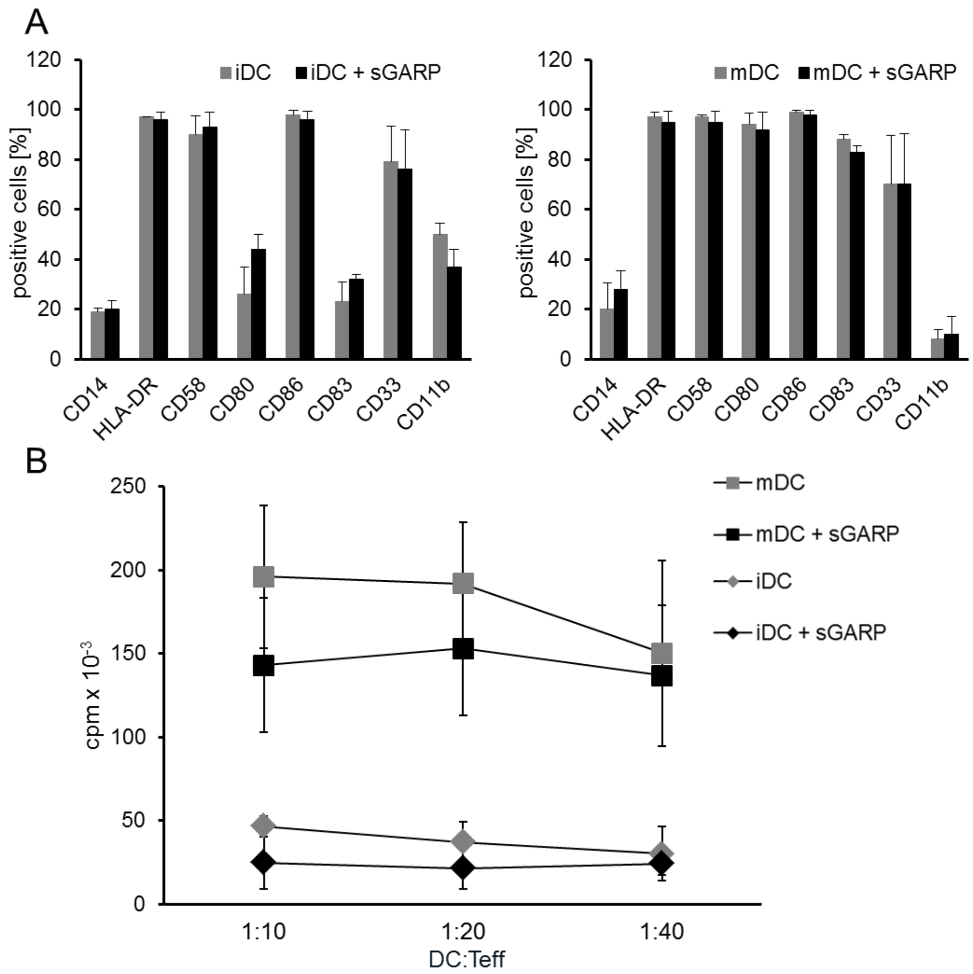

Supplemental Figure 2

Influence of sGARP on DC maturation and function (A) Effect of sGARP on DC maturation markers. iDC and mDC were cultured with the described cytokine cocktails with or without sGARP for the two last days of culture. Bar diagrams show marker expression on iDC and mDC (n=3, means ± SD). (B) T cell stimulatory capacity of iDC and mDC in the presence of sGARP. $CD4^+$ T cells were stimulated with allogenic iDC or mDC treated with/without sGARP at different DC:Teff ratios. Teff proliferation was determined on day 4 of culture by incorporation of ^{3}H-Tdr and is presented as means ± SD of triplicates of three independent experiments.

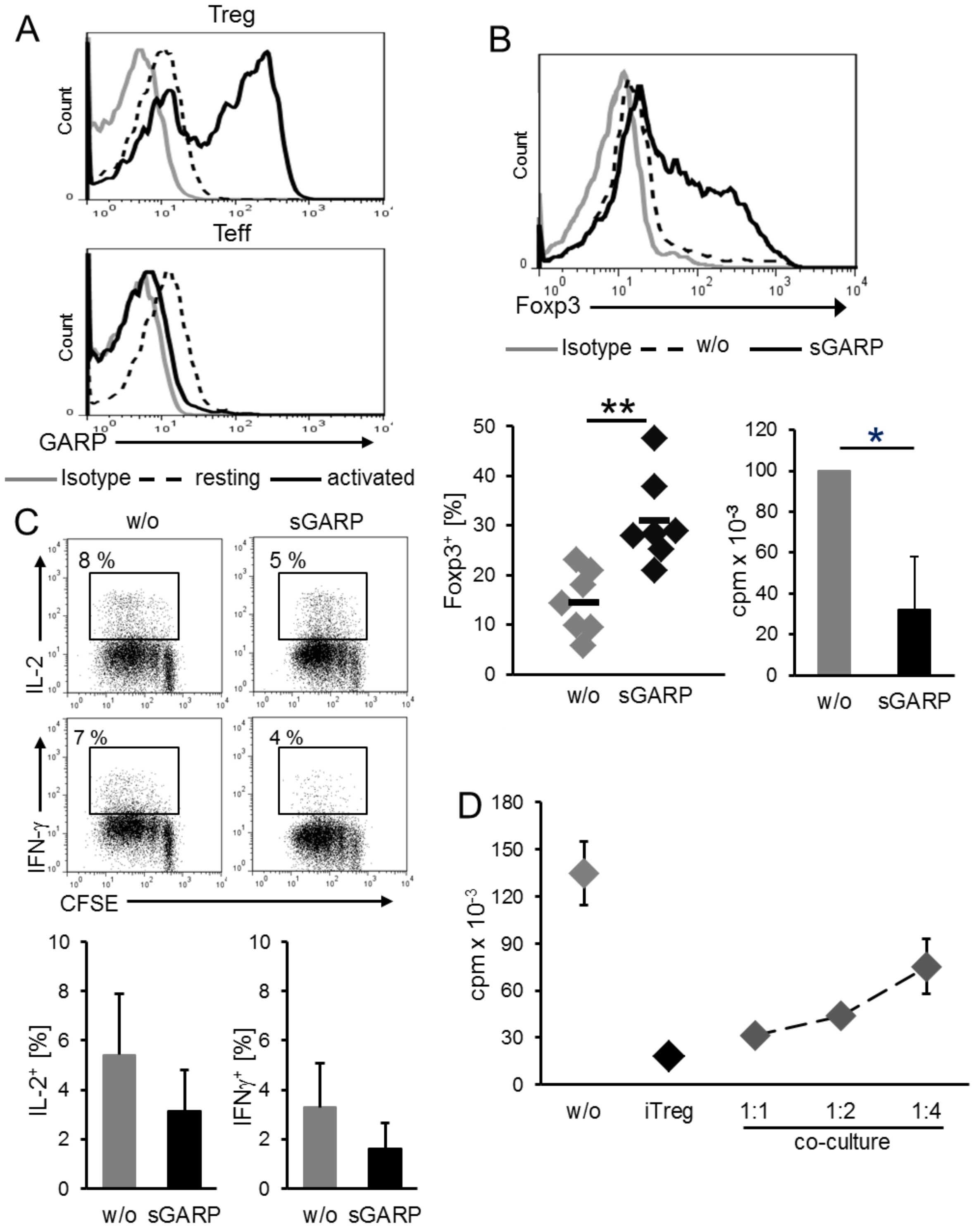

Supplemental Figure 3

(A) GARP is an activation marker on Treg. GARP expression on resting or activated (16 hours) CD4$^+$CD25$^+$ Treg and CD4$^+$CD25$^-$ Teff. Graphs show one representative experiment out of four. (B) sGARP increases Foxp3 expression and inhibits

proliferation. $CD4^+CD45RA^+$ T cells were stimulated with anti-CD3 (0.5 µg/ml) and anti-CD28 mAb (1 µg/ml) in the presence of sGARP (10 µg/ml). Foxp3 expression was analyzed on day 3 by flow cytometry. Graph shows one representative result (upper part). Diagrams (lower part, left) display summarized data of 7 independent experiments (means ± SEM, **$P<0.01$). Proliferation was analyzed on day 12 by ^{3}H-TdR incorporation (lower part, right). Bar diagrams show pooled data of 3 independent experiments (percentage of proliferation in the presence of GARP normalized to proliferation of cells without GARP; n = 3, means ± SEM, *$P<0.05$). (C) CFSE-labeled $CD4^+CD45RA^+$ T cells were stimulated with anti-CD3 and anti-CD28 mAb in the presence or absence of GARP (10 µg/ml). Cytokines were analyzed on day 10 upon re-stimulation. Dot blots and diagrams show one representative result of 5. (D) GARP-mediated Treg induction. Naive $CD4^+$ T cells (Donor 1) were stimulated with anti-CD3 and anti-CD28 mAb and cultured in the presence of GARP (10 µg/ml) for 7 days before being used as suppressor cells in a co-culture experiment. Allogeneic $CD4^+$ T cells (Donor 2) simultaneously stimulated for 7 days with anti-CD3 and anti-CD28 mAb served as responder cells. Co-cultures (10^5 responder cells, Donor 2) plus titrated suppressor cells (Donor 1) were stimulated with 0.5 mg/ml anti-CD3 mAb in the presence of 3×10^5 irradiated T cell–depleted PBMC feeder cells (Donor 3). Proliferation was determined on day 4 by a 16-hour ^{3}H TdR pulse. Results show the pooled data of 3 independent experiments (means ± SEM).
